# How common is chronic fatigue syndrome; how long is a piece of string?

**DOI:** 10.1186/1478-7954-5-6

**Published:** 2007-06-08

**Authors:** Peter D White

**Affiliations:** 1 Centre for Psychiatry, Wolfson Institute of Preventive Medicine, Barts and the London, Queen Mary School of Medicine and Dentistry, London, EC1A 7BE, UK

## Abstract

Commentary on

Prevalence of chronic fatigue syndrome in metropolitan, urban, and rural Georgia

William C Reeves, James F Jones, Elizabeth Maloney, Christine Heim, David C Hoaglin, Roumiana S Boneva, Marjorie Morrissey and Rebecca Devlin

## 

One of the most difficult tasks in medicine is to accurately measure how common illnesses are. Why do we do it? Justifications include being able to plan health care and public health priorities, as well as highlighting specific diseases for extra funding for both health care and research. Yet the jobbing physician at the sharp edge of clinical practice cares little about the exact prevalence of a disease or illness, since this is all too obvious from the frequency of the problems presented by patients who come through the door.

## How do you measure a syndrome?

If the disease in question has no biological marker and is difficult to define clinically, the problem of working out the accurate prevalence becomes esoteric. Chronic fatigue syndrome (CFS) is just such an illness. It has as many synonyms as putative causes, being also called myalgic encephalomyelitis, chronic immune dysfunction syndrome, and post-viral fatigue syndrome, amongst others. Since fatigue is one of the most common symptoms reported by patients in general, delineating a specific syndrome with fatigue as a central feature risks arbitrary decisions about ascertainment. Do we categorise the syndrome on the basis of the severity of fatigue, the number of associated symptoms, or the severity of the resultant disability? Even measuring the consequent disability gives us problems since there are only weak correlations between subjective and objective observations [1]. It is therefore no great surprise that half of all doctors do not even believe it exists [2].

And yet, patients and their organisations constantly criticize doctors both for not believing in the existence of CFS and for not taking patients seriously. Even politicians seem to take the problem more seriously than some doctors do. This may be as much to do with successful lobbying as the economic costs of CFS, which have been estimated as $9 billion per annum just for lost productivity in the USA [3]. Doctors don't understand things they can't see or measure, and patients mistrust doctors who don't understand them. We are in a conundrum.

## One way forward

The Centers for Disease Control and Prevention (CDC) in the United States of America are one of the few health care agencies who do take CFS seriously, to the extent of supporting a $4 million public education campaign, which started last year [4]. They have also led the way in providing operationalised criteria in order to standardize the diagnosis of CFS [5]. Their latest research programme has been based on a large survey of the adult (18–59 years old) population of the state of Georgia, USA, in order to better understand the epidemiology and etiology of CFS [6]. Their previous study of prevalence, in Wichita, Kansas, suggested a prevalence of 0.24% [7]. Another independent population survey in Chicago suggested a prevalence of 0.42% [8]. The CDC has now repeated and extended the Wichita study in Georgia, and found a prevalence of between six and ten times greater, with 2.5% of the population suffering from CFS [6]. If this prevalence was both accurate and representative of the USA as a whole, this would suggest that some 7.5 million Americans were sufferers, compared to the previous estimates of 0.7 to 1.2 million.

## A cautious interpretation

Could this really be true? The authors are sensibly cautious in their interpretations, and point out the uncertainties inherent within the study. There are three main reasons why we should be cautious about interpretation and generalizing from this finding. Compared to previous studies, there were important differences in the method of ascertainment used in the Georgia study that may help to explain the greater prevalence. Most importantly, the Georgia study used a different initial screening question. Instead of asking whether a household member was suffering from "fatigue", as previously done, the screening question asked about being "unwell", by which was meant having one or more of the following symptoms for a month or more: "fatigue, cognitive impairment, unrefreshing sleep, muscle pain, joint pain, sore throat, tender lymph nodes, or headache" (all being likely symptoms of CFS). The authors suggested that this stratagem picked up an extra 11.5% of CFS cases. A strength of the Georgia survey was the use of standardised measures of symptoms and disability. However in order to count someone as fatigued – the central criterion for a diagnosis of CFS – individuals only needed to score the median or more of the well population, either for fatigue or inactivity. In a previous study, the same authors found that using such standardised measures picked up three times as many cases of CFS than verbatim enquiries [9]. These methodological differences mean it is not possible to directly compare the prevalence of CFS in Georgia with previous studies.

Comorbid psychiatric conditions may have inflated the prevalence. A previous study found an equally high point prevalence of CFS (2.6%), by surveying United Kingdom primary care patients [10]. However, when those patients who also had a comorbid psychiatric disorder were excluded, the prevalence fell to 0.5%. Although it will be important to publish the prevalence of comorbid psychiatric disorders in the Georgian survey, the argument can still be put that these comorbid psychiatric disorders were secondary to having chronic ill-health, rather than the primary and explanatory condition. The current design cannot determine the direction of causality, although previous longitudinal studies suggest that psychiatric ill health can both follow and precede CFS [11,12].

Georgia may not be representative of the USA as a whole. For instance, we do not know the body mass index (BMI) of the Georgian sample. The Wichita sample of CFS cases contained 43% of subjects with a BMI of 30 or over, representing significant obesity [9]. This compares with 20% in the USA as a whole [13]. Since obesity is associated with fatigue [14], a similar proportion in Georgia might inflate the prevalence of CFS.

## To conclude

What can we conclude from this very large survey? Although methodological issues may help to explain the high prevalence of CFS found in this study, the argument can still be made that the prevalence of CFS is greater than previously thought [10,15]. CFS is at least as common in ethnic minorities in the USA as in the ethnic Caucasian majority; a welcome replication of previous studies [8]. CFS is not an exclusively white syndrome. Social issues may help to explain why women suffer CFS more than men. But perhaps the most important conclusion is that there were about twice as many people in Georgia who were unwell with fatigue, who did not meet the criteria for CFS. Our current criteria for diagnosing CFS are arbitrary [16], and we need to widen the net to capture all those people who become so chronically tired and unwell that they can't live their lives to their full potential. The jobbing physician does not close the door on those who don't meet criteria.

## Conflict of interest

Professor White has collaborated with some of the authors in research into CFS.
